# Conferences/Events: U.S.-JAPAN PANEL ON WIND AND SEISMIC EFFECTS National Bureau of Standards, Gaithersburg, MD, May 17–20, 1988

**DOI:** 10.6028/jres.093.162

**Published:** 1988-12-01

**Authors:** Noel J. Raufaste

**Affiliations:** Center for Building Technology, National Engineering Laboratory, National Institute of Standards and Technology, Gaithersburg, MD 20899

The U.S.-Japan Panel on Wind and Seismic Effects provides for cooperative activities of 15 U.S. and 6 Japanese Agencies to reduce damage from strong winds and earthquakes. The Panel is part of the U.S.-Japan Natural Resources Development Program under the aegis of the U.S.-Japan Cooperative Science Program of 1961. The National Bureau of Standards (NBS) provides the U.S.-side chairman and secretariat; Dr. Richard N. Wright, Director of the Center for Building Technology (CBT) and Noel J. Raufaste of CBT, respectively. Annually, the Panel conducts a 1-week joint technical meeting where recent research results are discussed. A technical study tour follows the Panel meetings.

This year (1988) the United States hosted the 20th joint meeting and visits to U.S. laboratories and construction projects. The meeting was held at NBS during 17–20 May. Technical presentations focused on: Wind Engineering; Earthquake Engineering; Storm Surge; Tsunamis; U.S.-Japan Cooperative Research Program (where NBS is performing cooperative research in seismic behavior of large concrete bridge piers); summaries of recent Panel Workshops; and a report from each side on Two Decades of Panel Accomplishments and Future Work.

During 1988–89, the Panel members agreed to performing large-scale testing of precast seismic structural systems, advance technologies in application of active and passive control of buildings and other structures, improve knowledge of strong motion data on performance of buried pipeline systems, and verify the effectiveness of retrofitting and strengthening methods for structures and soils. Five workshops are being planned on: repair and retrofit of buildings, sensor technology applied to large engineered systems, earthquake hazard and risk assessment, disaster prevention for lifeline systems, and loads on bridges. Also, a planning meeting will address remedial treatment of liquefiable soils.

Panel accomplishments during the past year included: more than six guest researchers exchanged between U.S. and Japanese laboratories; correlation of data from the Whittier Narrows and the Mexico City earthquakes with Japanese and U.S. design practices; joint research in large-scale testing of masonry and lifeline structures; sharing repair methods for civil engineering structures; exchanged data from strong motion arrays to study causes and effects of horizontal variations in ground motions and effects of overburden on intensity of ground shaking; and translated into English and published the Japanese publication, *Manual for Repair Methods of Civil Engineering Structures Damaged by Earthquakes.* This report is a valuable reference for development of U.S. building practices. The data produced by the joint Panel influence on-going U.S. and Japan structural engineering research, and guide improvements of building codes and standards of both countries.

